# Exploring exhaled breath biomarkers for lactose intolerance diagnosis: the Lactobreath pilot study protocol

**DOI:** 10.1136/bmjopen-2025-107256

**Published:** 2025-08-03

**Authors:** Stamatios Giannoukos, Kathryn Jane Burton-Pimentel, Roxane Guillod, Guy Vergères, Daniel Pohl

**Affiliations:** 1 Department of Chemistry and Applied Biosciences, ETH Zürich, Zürich, Switzerland; 2 Agroscope, Bern, Switzerland; 3 aha! Swiss Allergy Centre, Bern, Switzerland; 4 Department of Gastroenterology and Hepatology, USZ, Zürich, Switzerland

**Keywords:** Microbiota, Nutritional support, NUTRITION & DIETETICS, Gastrointestinal Microbiome

## Abstract

**Introduction:**

Food intolerances are prevalent in Europe and can cause considerable physical discomfort, dietary restrictions and psychosocial challenges. Among the prominent causes of food intolerance are defects in the digestion and/or transport of short-chain fermentable carbohydrates, fermentable oligo-, di-, monosaccharides and polyols (FODMAPs). A common diagnostic tool for food intolerance is the hydrogen breath test, which monitors the production of H_2_ gas from the fermentation of ingested FODMAPs by colonic microbiota. However, this method is limited due to its relatively poor correlation with gastrointestinal (GI) symptoms experienced by patients. Diagnosis is complicated as food intolerance is often associated with functional GI disorders, while FODMAPs may exert their effects individually or in combination. Further research on the pathophysiology and the impact of intervention strategies for these conditions is required to improve the diagnosis of food intolerance.

**Methods and analyses:**

The Lactobreath pilot study is a randomised, two-arm, double-blinded controlled study. 120 healthy, free-living adults will undergo 6-hour postprandial tests with lactose or glucose (control) to investigate the molecular composition of human exhaled breath (exhalome) as a potential source of biomarkers associated with clinical and metabolic traits of lactose malabsorption (Lactobreath profiles). This serves as a proof-of-concept for the future application of this technology in diagnosing food intolerance. We will use a sensitive, non-invasive, real-time measurement technique based on secondary electrospray ionisation coupled with high-resolution mass spectrometry to analyse the chemical profile of the postprandial exhalome after lactose ingestion. Symptoms of lactose intolerance will be assessed using a standardised questionnaire and mechanistically linked to specific key metabolites of the discriminating breath profile. In parallel, a solid-state sensor will measure postprandial hydrogen gas in breath samples, while GI gases (CH_4_, H_2_, O_2_) and intestinal transit time will be monitored using a novel ingestible gas sensor (Atmo Gas capsule). Metabolites in urine, including lactose-derived metabolites, will be investigated using gas chromatography coupled with mass spectrometry. Postprandial bowel sounds will be recorded by wearable sensors (DigeHealth AG). Baseline assessments will be completed before the dietary challenge to capture usual dietary intake (repeated 24-hour recall), faecal microbiota (shallow shotgun sequencing) and to evaluate genetic polymorphisms using saliva samples (PCR analysis of selected penetrant single-nucleotide polymorphisms).

**Ethics and dissemination:**

The Lactobreath study has been approved by the Ethics Committee of the Canton of Zurich, Switzerland (#2023-01639). The project results will be published in open-access journals, presented at national and international conferences and communicated to the public and other relevant stakeholders via the communication channels of all investigators and partners. All results derived from the study will be accessible, in line with the Swiss National Science Foundation open access policy.

**Trial registration number:**

NCT06177938.

**STRENGTHS AND LIMITATIONS OF THIS STUDY**

- Advancement of high-resolution metabolomics to explore the interface between food intake and human physiology through breath analysis.
- Controlled study design to limit variation in metabolite levels due to endogenous or external factors (eg, usual dietary intake, circadian rhythm, medications, cosmetics, exercise).
- Strict inclusion criteria and study demands may limit participant recruitment and retention.
- Metabolite identification is complex due to ambiguous ion assignments; this is addressed via a comprehensive annotation strategy using exhaled breath condensate collection, pathway analysis, standards and multiple analytical platforms.

## Introduction

 Food intolerance affects a significant proportion of the population, provoking gastrointestinal (GI) symptoms, including increased flatulence, abdominal pain, bloating and diarrhoea.^1^ Defects in the digestion and/or transport of short-chain fermentable carbohydrates, referred to as fermentable oligo-, di-, monosaccharides and polyols (FODMAPs), including lactose and fructose, are among the prominent causes of food intolerance. The pragmatic way to diagnose food intolerance is to confirm the absence of organic diseases or food allergies, before proceeding to the exclusion of FODMAPs from the diet of patients, followed by their controlled individual reintroduction.^1 2^ Diagnostic tests, such as the H_2_ breath test that monitors the production of hydrogen gas by the colonic microbiota fermenting the FODMAPs, have methodological limitations and do not consistently predict the GI symptoms experienced by the patients,^1^ reflecting the complexity of the mechanisms involved in food intolerance. Indeed, although the increased small intestinal water content due to the osmotic activity of the ingested carbohydrates is known to induce diarrhoea, and bacterial production of gases is a causal factor of flatulence and bloating, other factors can influence the GI symptoms associated with food intolerance, including colorectal transit time, the composition of the gut microbiota and immunologic phenomena.^2^ Interestingly, volatile compounds present in faecal water have been associated with GI symptoms of food intolerance.^3^ Furthermore, FODMAPs may exert their effect individually or in combination, further complicating food intolerance diagnosis and management. Managing food intolerances by low FODMAP diets is also complicated because the complete elimination of food sources of FODMAPs may induce nutritional deficiencies in patients. Reintroducing tolerated doses of the incriminating FODMAPs is, therefore, an integral part of the management of food intolerance.^4^,^7^

Research on the pathophysiological aspects of these conditions and the efficacy of intervention strategies is required to support the diagnosis and management of food intolerance.^6^ Furthermore, tests that can predict patients’ response to low FODMAP diets are also needed.

The Lactobreath study will investigate lactose intolerance (LI) in a proof-of-concept study to demonstrate the potential of exhaled breath as a source of biomarkers associated with food intolerance. LI describes the clinical symptoms resulting from lactose malabsorption (LM), the failure to digest and/or absorb lactose in the small intestine. In infants, the disaccharide lactose that is present in milk is cleaved in the small intestine by lactase, an enzyme encoded by the *LCT* gene, to produce galactose and glucose. However, lactase expression is epigenetically regulated and decreases during infancy in most humans.^8^ Polymorphic modifications associated with the development of pastoral activities approximately 8000–9000 years ago counter the epigenetic regulation of LCT so that about one-third of the human population now maintains an intestinal lactase activity into adulthood.^9^ Recent research has revealed that epigenetic regulation of lactase expression also occurs in lactase-persistent (LP) adults, explaining the decreased lactose-digestive ability observed with ageing.^8 9^ The consumption of lactose in the absence of intestinal lactase activity results in the lactose entering the colon, fermented by the resident microbiota, producing short-chain fatty acids (SCFAs) and gases such as H_2_, CH_4_ and CO_2_. The osmotic changes resulting from lactose in the colon, combined with these fermentation end-products, induce GI symptoms of LI, abdominal pain, bloating, flatulence, nausea and diarrhoea.

Various diagnostic approaches for assessing LM exist, including blood metabolite tests, breath measures, genetic tests and clinical presentation.^10^,^21^ While jejunal biopsies are considered the clinical gold standard for LM diagnosis, the lactose hydrogen breath test (LHBT) is the most widely used clinical diagnostic tool. The LHBT is based on measuring the H_2_ levels in the exhaled air of an individual for up to 5 hours postprandially after consumption of a specific dose of lactose. Diagnosis of LI is confirmed by the presence of H_2_, indicating that lactose has entered the large intestine and has been metabolised by the colonic microbiota, accompanied by GI symptoms. The available diagnostic tests are limited in their sensitivity and specificity, leading to frequent misdiagnosis and complicated by the presence of concurrent food intolerances. The medical domain has therefore moved to a clinically more relevant and inclusive approach by focusing diagnostic efforts on the GI symptoms of LI in the broader context of the FODMAPs.^1^ The Lactobreath study will focus on and investigate the potential of human exhaled breath as a source of biomarkers reporting on the clinical traits associated with LM, as a proof-of-concept for the diagnostic and dietary management of food intolerances.

Nutritional research has widely embraced metabolomic approaches to uncover the molecular complexity of foods and the consequent complexity of the response of organisms to food ingestion. Standard methodologies for monitoring the impact of food consumption on human metabolism, such as using food intake biomarkers combined with the measurement of validated clinical parameters, are often time-consuming or invasive, as they typically require human urine or blood analyses.^22^,^27^ In contrast, the analysis of exhaled breath presents an attractive alternative due to its non-invasive character, given the evidence for its high sensitivity demonstrated in extensive studies and applications over the last decades on various health-related issues.^2228^,^30^

Exhaled breath originates from the lungs and airways and contains a complex mixture of inorganic gases, traces of thousands of volatile organic compounds (VOCs) and microscopic aerosol particles.^31^,^38^ Both human breath VOCs and aerosol particles carry molecular information that comprises health and disease-related markers that can be used for medical diagnostic purposes. The number of clinical trials using breath analysis has experienced a steady rise over the last two decades,^39^ with wide applications in medical diagnostics, therapeutic monitoring and toxicology/precision medicine. Similar to blood, the composition of the exhaled breath in response to nutrition and dietary intake results from the metabolism of nutrients in the GI tract, the liver and the other body organs. This offers a unique opportunity to assess and understand the differences in individual responses to the same diet.

Recent advances in chemical sensing tools^40^,^43^ have facilitated the analysis, screening and decoding of volatile metabolites produced by the complex biochemical processes that evolve continuously within the human body. Existing mainstream technologies for the analysis of exhaled breath use either standalone or hyphenated mass spectrometry (MS) systems,^32 40^ ion mobility spectrometry,^44^,^48^ electronic noses^32^ and laser spectroscopy.^49 50^ Widely used off-line breath analysis techniques for VOCs and semi-VOCs include gas chromatography-mass spectrometry (GC-MS),^51^,^54^ GCxGC-MS^33^ and thermal desorption GC-MS,^55^ typically employing gas sampling bags, syringes or canisters. Proton transfer reaction MS,^56^,^61^ selected ion flow tube MS^61^,^65^ and membrane inlet MS^66 67^ serve for both online and offline analyses. Recently, high-resolution MS (Orbitrap, time-of-flight mass analysers^68^) coupled to ambient ionisation sources such as direct analysis in real-time,^69^ atmospheric pressure chemical ionisation^40^ and secondary electrospray ionisation (SESI)^70^,^78^ has been applied to measure human exhaled breath for disease diagnostic purposes in real-time. The development of real-time breath measurements offers a unique opportunity to assess the molecular breath signatures (exhalome) associated with the rapid onset of symptoms exhibited in food intolerance, a non-invasive strategy to gain insights into the specific metabolic response of individual consumers and offering a new route toward personalised nutrition.

The Lactobreath study uses state-of-the-art real-time breath metabolomics, suited for sensitive, non-invasive measurement of breath biomarkers in a controlled nutritional intervention that offers extensive and comprehensive participant characterisation for clinical and metabolic features relevant to lactose metabolism. A holistic approach is defined in the Lactobreath study to address and explore some of the challenges in the diagnosis of food intolerance by capturing metabolic processes and physiological variables that influence clinical phenotypes. Indeed, the Lactobreath breath profiles are focused on assessing clinical symptoms in the context of multiple existing diagnostic tools as well as physiological factors (eg, gut microbiota, GI transit) that are known to influence food intolerance symptoms. The Lactobreath study ultimately aims to support the development of a diagnostic test for LM and LI based on exhaled breath metabolomics, providing a non-invasive proxy for the subjective GI symptoms experienced by humans.

## Hypothesis and objectives

### Hypothesis

We hypothesise that lactose consumption generates different breath metabolomic profiles in people with lactose tolerance and those diagnosed with LI. These breath profiles are also expected to be associated with GI symptoms of LM.

The primary objective of the study is to identify postprandial metabolic profiles in human exhaled breath associated with GI symptoms of LM (Lactobreath profiles).

Secondary objectives:

- To identify postprandial metabolic profiles in human exhaled breath reporting on the clinical traits associated with LM (Lactobreath profiles), including:
  ▪ Genetic polymorphisms regulating the expression of the lactase gene.
  ▪ Breath hydrogen.
  ▪ Lactose-derived urinary metabolites.
- To mechanistically link Lactobreath profiles with metabolic traits associated with LM, including
  ▪ Colonic gases measured by the Atmo Gas Capsule.
  ▪ Urine metabolome.
  ▪ Gut microbiota composition.
- To associate colonic gases and symptoms of LI with bowel sounds using a digital biosensor.

## Methods and analysis

### Study design

The study design is a randomised, two-arm, double-blinded intervention. The study set-up is a monocentric, national study. The study design allows the discovery of biomarkers that report on LM by comparing the breath response to lactose ingestion in three predefined groups defined by the characteristics of LM. The study began in June 2024, with an expected primary completion by the end of 2025.

The specificity of the candidate biomarkers to lactose exposure will be confirmed by the responses of the biomarker in the control condition (glucose solution). By randomising the participants to lactose or glucose intervention arms, the risk of bias due to unknown confounding factors is reduced. Furthermore, the participants will be blinded to the intervention type received to limit non-metabolic responses to dietary exposure. There is a risk that by using a parallel study design, some variation in the biomarkers that relate to the individual will be introduced; however, for the purpose of diagnostic testing, the biomarkers need to be highly specific and robust to variation between individuals. Consequently, the choice of design could help identify more discriminant biomarkers.

### Study population and eligibility

The study will be conducted in healthy adult men and women with or without the genetic ability to digest lactose. Participants will be enrolled according to inclusion and exclusion criteria based on those described by Ramakrishnan *et al*,^79^ ensuring the selection of participants with homogenous clinical profiles and avoiding pathologies that could confound the study results. Specifically, eligible adults (aged 18–65 years at screening) must reside in Switzerland, be English/German speakers and agree to refrain from all other treatments and products used for dairy intolerance (eg, Lactaid dietary supplements) during study involvement.

Study exclusion criteria include the following: milk allergy, pregnancy or lactation at time of enrolment, cigarette smoking or other use of tobacco or nicotine-containing products (<3 months of screening), diagnosis with disorders known to be associated with abnormal GI motility, history of surgery that alters normal GI tract function, suspected obscure GI bleeding, diabetes mellitus, HIV, hepatitis B or hepatitis C, body mass index >35 kg/m^2^, chronic antacid and/or proton pump inhibitor use, recent use of systemic antibiotics (<2 months of screening), history of alcohol and/or drug abuse (past 12 months), recent bowel preparation for endoscopic or radiologic investigation (<4 weeks of screening), severe irritable bowel syndrome (IBS) (ie, IBS Symptom Severity Score >400), dietary restrictions including vegan or vegetarian diet and enrolment in another clinical trial (<3 months). In addition, exclusion criteria for using the Atmo Gas Capsule are applied to minimise the risks associated with its use: presence of strictures (suspected or known), fistulas or any GI obstruction; gastroparesis; history of gastric bezoar; swallowing disorders or dysphagia to food or pills; presence of implantable or portable electromechanical medical devices (eg, pacemakers). All participants must provide informed, written consent for participation in the study. Non-completion of the study procedures is an exclusion criterion.

A total of 120 adult participants will be selected and distributed into three groups based on the results of a screening test to confirm their genetic LM profiles and response to the ingestion of 25 g of lactose (lactose solution) test:^17^

- 24 genetically LP subjects with no GI symptoms in response to the ingestion of lactose (group 1).
- 24 genetically lactase-non-persistent (LNP) subjects with no GI symptoms in response to the ingestion of lactose (group 2).
- 72 genetically LNP subjects with GI symptoms in response to lactose ingestion (group 3).

Participants confirmed as genetically LP but experiencing GI symptoms after ingesting lactose will be excluded, as factors other than primary hypolactasia likely explain their symptoms.

The groups are chosen to allow discrimination of metabolic signals that relate to the genetic and clinical phenotypes of LI. Group 3 intentionally comprises more individuals to allow for secondary analysis of the differences in symptom severity, which is often very variable. The groups are important to enable the detection of biomarkers sensitive to differences in symptoms of LI to be captured.

### Recruitment, screening and informed consent procedure

Participants will be recruited by adverts distributed via the communication channels of the project partners (social media, newsletters, webpages, clinical locations, etc). Telephone and email screening will be used to identify candidate participants and to inform interested participants of the study details. An inclusion visit at the Department of Chemistry and Applied Biosciences at ETHZ will be set up for interested candidates. During the inclusion visit, participants will receive comprehensive information about the study and conditions of participation, permitting informed consent to be given. Inclusion and exclusion criteria will be assessed according to a structured interview and confirmed by a medical doctor.

### Genetic and lactose tolerance screening

For all eligible participants, a home screening test will be conducted after consent is obtained. The home screening test kit will contain lactose powder and accompanying information so that the subjects can conduct a lactose tolerance test (according to the LHBT protocol) at their home. The test will be conducted based on a standardised, previously validated questionnaire,^17^ adapted to the most recent guidelines (https://www.esnm.eu/guidelines.html)^80^,^82^ that propose the use of 25 g of lactose for lactose tolerance testing. Briefly, abdominal pain, nausea, bloating, diarrhoea, borborygmi, as well as the sum of all symptoms, will be measured on a 10-point scale with symptom intensities ranging between 0 and 9. The home screening test kit will also provide material to collect a buccal sample to determine genetic polymorphisms commonly modulating LP.

From the participants who return the buccal samples and whose polymorphism for LP is measured, 120 participants will be selected and distributed into three groups based on their LP genetic profiles and symptomatic response to the ingestion of 25 g of lactose (lactose solution).

LP volunteers experiencing symptoms will be excluded as factors other than primary hypolactasia likely explain their symptoms.

### Consent procedure

A participant’s formal written consent (online supplemental file 1) will be obtained before the participant is submitted to any study procedure. The participant is not obliged to give consent during the inclusion visit, but the screening may not take place without it. The consent form will be signed and dated by the investigator or his designee at the same time as the participant’s consent is obtained. The study participant will receive a copy of the signed informed consent, which will be retained as part of the study records.

### Study intervention

The study intervention is a carbohydrate challenge (lactose or glucose) to identify postprandial metabolomic profiles in human exhaled breath that report symptoms of LI. Participants will be assigned to one of the challenges, using stratified randomisation to ensure a balanced distribution of participants with different tolerances to lactose consumption (based on phenotype and genotype). The glucose challenge will serve as a control and enable us to consider the specificity of the Lactobreath profiles for lactose metabolism. Both challenges consist of the ingestion of the carbohydrate solution (25 g of lactose or equimolar amounts of glucose: 13 g glucose^83^) dissolved in 150 mL of water. Bias related to an expected response to consuming lactose is limited by using a double-blinded protocol for the carbohydrate intervention with a control test (glucose) to confirm the specificity of any results observed for the lactose intervention. The participants’ response will be monitored with non-invasive sampling (ie, breath and urine), wearable devices for assessment of bowel sounds and symptom assessment for 6 hours after the ingestion of the assigned carbohydrate. Intestinal gases will be monitored during the challenge by a commercially available gas-sensing capsule that will be ingested within 5 min of the ingestion of the carbohydrate solution. All experiments will be conducted at the breath analysis laboratory at the Department of Chemistry and Applied Biosciences at ETHZ.

### Study procedures

**Figure 1:**
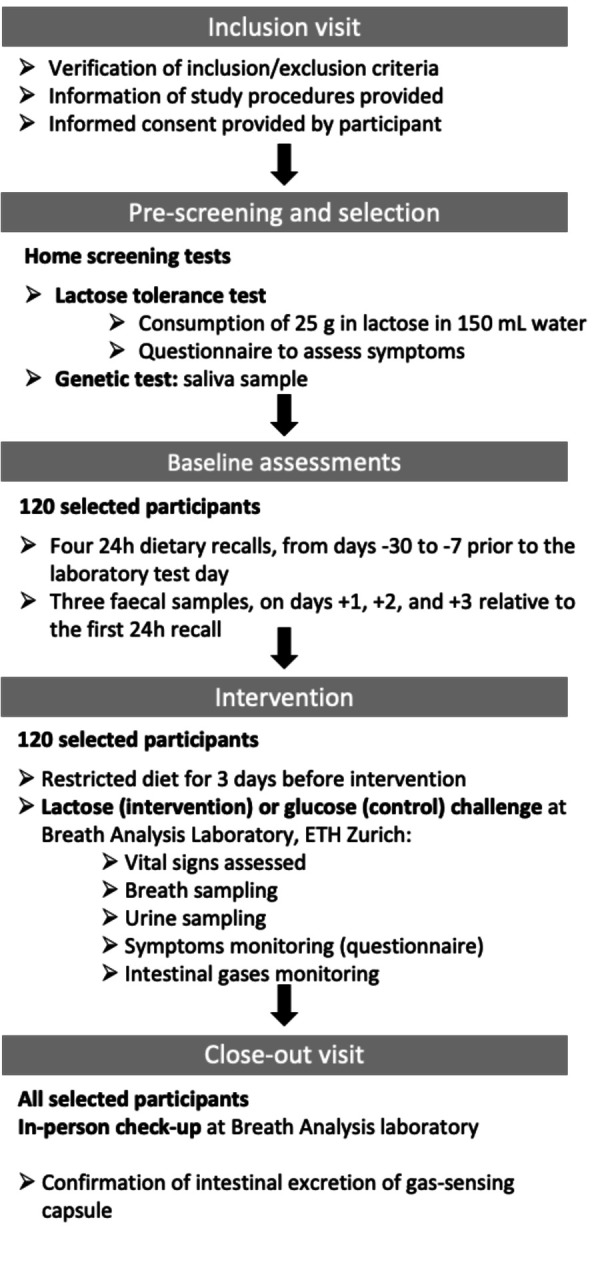
Overview of the planned project study protocol (screening, assessment of the baseline, intervention and completion of the study).

For each subject, baseline measurements will be made within 4 weeks of the intervention day which is followed by a close-out visit. The schedule of assessment table (figure 1) provides an overview of the study visits, procedures and samplings.

#### Baseline assessments

The assessment of diet and faecal microbiota will be coordinated and repeated, given the importance of obtaining robust baseline values in studies undertaking diet-microbiome analyses and the high inter-diurnal variation associated with these methods (within-person random error):^84^

- Four 24-hour dietary recalls will be completed using the myfood24 online platform on four consecutive days, from days −30 to −7, before the laboratory test day.
- Three faecal samples will be collected by the participants at home, ideally on three consecutive days, on days +1, +2 and +3 relative to the first 24-hour dietary recall.^85^

#### Intervention day

The diet of all selected participants will be restricted for 3 days before the carbohydrate challenge test day to ensure that all FODMAPs are avoided. A standardised diet providing all meals to the participants for 3 days before the test.

**Figure 2:**
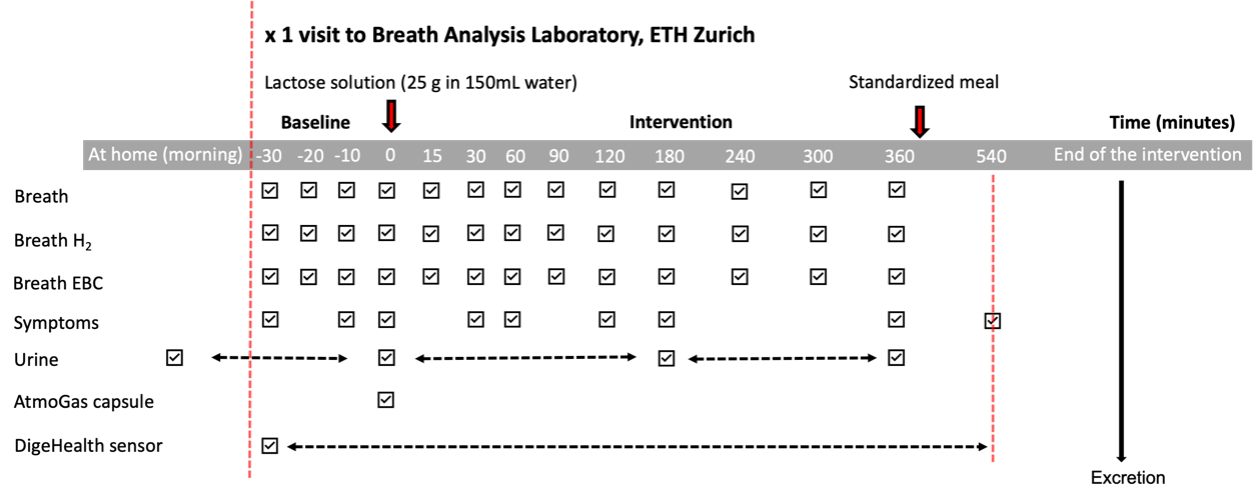
Overview of the planned intervention sampling protocol at the study site. EBC, exhaled breath condensate.

On the morning of the test day, the participants will be asked to collect their first test sample, the morning urine and consume 300 mL of water at home. On arrival in the test laboratory (Department of Chemistry and Applied Biosciences at ETHZ), the participants will deliver further samples for baseline measures (figure 2):

- Second urine sample.
- Breath for measure of exhaled H_2_ at times −30 min, −20 min and −10 min.
- Breath for the measure of exhaled metabolome at times −30 min, −20 min and −10 min.

The urine samples will be used for metabolomics analysis and, in the case of female participants, for a pregnancy test. Participants with a positive pregnancy test will be excluded.

All measures are indicated with respect to the start of the test (ie, the start of the consumption of the assigned carbohydrate solution), defined as t_0_.

In addition, the participants will fill out the questionnaire on LI (as used for the screening survey) at time −30 and −10 min to describe symptoms of LI before the test (baseline symptoms).

Thirty minutes before the start of the test, participants will be instructed to affix two DigeHealth wearable devices around their abdomen to record bowel sounds: one in the upper left abdominal region and another in the lower right abdominal region.

The participants will be assigned to one of the two carbohydrate challenges using stratified randomisation based on the three groups defined by the pre-screening tests. The randomisation is defined using a web-based randomisation procedure, the Clinical Trial Randomization Tool provided by the National Cancer Institute’s Division of Cancer Prevention (https://ctrandomization.cancer.gov). 100 participants will be assigned to the lactose group and asked to ingest 25 g of lactose dissolved in 150 mL of water. The remaining 20 participants (4 participants from each group 1 and 2, and 12 participants from group 3) will be assigned to the glucose group and asked to ingest equimolar amounts of glucose (13 g)^83^ dissolved in 150 mL of water. The participants and investigators will be blinded during testing to the type of carbohydrate solution assigned. Measurement bias will be addressed for all analytical platforms by measuring the samples without removing the blinding.

For each participant, the carbohydrate solution must be consumed in a time lapse of 5 min. Following the solution intake, participants will be asked to rinse their mouths with a standard quantity of water (500 mL) to avoid the detection of solution-relevant residual molecules in the oral cavity.

The participants will swallow a commercially available gas-sensing capsule (Atmo Gas Capsule) within 5 min following the lactose or glucose solution intake. The gas-sensing capsule will measure intestinal gases (hydrogen, oxygen and carbon dioxide) during its transit through the GI tract and allow calculation of the small intestinal and colonic transit times and regional gut fermentation patterns. Ingestible sensors are usually ingested with a meal to locally report on the physiological properties of the GI tract during meal processing. In this study, the gas-sensing capsule will be ingested with a carbohydrate solution, and thus it will likely not follow the same kinetics as the lactose metabolism. However, the capsule monitors gaseous profiles that provide information on its GI localisation and the production of lactose-derived H_2_ by the colon processes over several hours after its ingestion; thus, a definition of the time window during which the gas-sensing capsule will report on lactose processing will be possible. Preprandial and postprandial samples will be collected at predefined times as indicated in figure 2.

To normalise participants’ hydration, particularly to facilitate urine collection, they will be offered a standardised quantity of water based on their body weight to be consumed in regular portions during the post-ingestion period. No other foods or fluids will be permitted during the 6-hour postprandial testing.

After the 6-hour sampling, the participants will be given a standardised meal at the laboratory. Following this meal, they may drink still water ad libitum but will refrain from eating other foods until the final symptoms assessment 9 hours after the intervention.

Intestinal excretion of the gas-sensing capsule will mark the end of the intervention. This will be confirmed by participant verification of the mobile phone app each bowel movement to check if: (a) the temperature has dropped below 33°C and/or (b) the data transmission after flushing away the faeces is lost. In the time following the carbohydrate challenge and before excretion is confirmed, the participant can resume their everyday life while always keeping the capsule receiver within 2 m of their body. The participant should avoid strenuous exercise before the capsule is excreted. Before the confirmed excretion of the capsule, participants must not undergo an MRI examination unless an X-ray is performed to confirm that the capsule has left the body. If the excretion of the capsule is not confirmed, the recommended Capsule Retention Procedure, defined for the use of Atmo Gas Capsule in previous clinical trials, will be implemented by the team with the gastroenterology team of UHZ. The bowel sound wearable devices should be kept on for 9 hours after the beginning of the test until the final symptoms assessment 9 hours after the intervention.

Additional subjects will be recruited from the pool of participants to the survey should the number of participants completing the lactose and glucose tests fall below the targeted numbers. It should be noted that although symptoms of small intestinal bacterial overgrowth might be experienced by some participants after glucose ingestion,^85^ the acute nature of the study design renders this possibility unlikely. These participants will be retained for the data analysis, and their parameters, including the GI symptoms, will be analysed in light of the composition of their faecal microbiota.

### Sample collection

During the baseline period, all participants will collect and send by post stool samples for genomic characterisation of the gut microbiota. Faecal samples will be stored at −80°C. They will be analysed using shallow shotgun sequencing for a mechanistic understanding of the microbial features associated with lactose metabolism.

Breath and urine will be sampled on the intervention day, both before and during the 6 hours following the consumption of the assigned test drink. Breath sampling will be performed using a secondary electrospray ionisation source coupled to a high-resolution mass spectrometry system for direct and real-time detection of VOCs and using an H_2_ breath solid-state sensor for detection of exhaled hydrogen. Standardised breath sampling protocols will be developed for precise measurements of biomarkers of dietary intake and metabolism.

In addition, exhaled breath condensate (EBC) samples will be collected using a glass cold trap cooled to −78°C using a mixture of isopropanol. To collect EBC samples, participants exhale through the spirometry filter and a short-length tube, and their exhaled breath condenses in the cold trap. Blank samples will also be obtained by flushing 2 mL of water through the tubing and freezing it in the cold trap. All EBC samples will be stored at −80°C at the premises of ETHZ until thawed for subsequent analysis. Participants will be provided with the needed breath delivery equipment (ie, mouthpiece) and asked to breathe normally for up to twelve times per sample (this includes baseline measurements). Breath samples will be collected before the intervention and at nine time points following the intervention (15 min, 30 min, 60 min, 90 min, 120 min, 180 min, 240 min, 300 min and 360 min). Participants will be asked to provide four urine samples (two baseline samples before the intervention and two sample pools collected during the intervention (0–180 min and 180–360 min). Samples will be stored at 4°C during the test and before further analysis, all urine samples will be stored at −80°C at ETHZ.

### Safety

The main risk presented by the study intervention (ingestion of lactose solution) is minor GI symptoms (eg, abdominal discomfort) due to LM, and does not differ from the widely used LI diagnostic protocol, the LHBT. There is a potential risk associated with the use of a gas-sensing capsule for the investigation: the failure of the capsule to be excreted. A protocol is in place for this very unlikely eventuality that will be implemented under the guidance of the study Principal Investigator (PI) and a specialist gastroenterology team of the University Hospital Zurich. No cases of capsule retention have been reported for the Atmo Gas Capsule across the various clinical trials conducted for the product to date. The risks of the intervention are outweighed by the potential benefits of the study for improving clinical diagnosis of food intolerances (improved specificity of food intolerance diagnosis using non-invasive breath measurements) and offering mechanistic insights into the symptoms associated with LM. No severe adverse reactions are anticipated, but the Monitoring Committee will check for these.

### Analysis of samples

#### Real-time breath measurements (breath metabolome)

On-line chemical analysis of exhaled breath will be carried out by ETHZ using a SESI source purchased from Fossil Ion Technology SL, coupled with an HR-MS system (Q Exactive Orbitrap, ThermoFisher Scientific). SESI-MS is a well-established and robust analytical technology specially developed for in-depth breath characterisation. Its applicability has already been demonstrated in clinical studies previously published by ETHZ.^70^,^77^ The real-time approach of this method is practical for the high sampling frequency planned for the study and has already been shown to be sensitive to acute dietary intervention.^78^

By conducting breath analysis during the 6 hours following the intervention, it will be possible to follow multiple features over time and to obtain a major part of the postprandial kinetic profile of the participants. During a real-time breath measurement using SESI-MS, the participant exhales through a disposable antibacterial mouthpiece and a short-length heated sample transfer line into the ionisation chamber. In the SESI chamber, a mist of charged droplets interacts with breath molecules for charge transfer and ionisation. The produced charged breath molecules are then introduced into the mass analyser for mass-to-charge (m/z) separation and detection. Measurements are carried out in the positive and negative ion mode that facilitates the determination of protonated and deprotonated species, respectively. Exhaled CO_2_, pressure, flow rate and breath volume are simultaneously recorded using an interface (EXHALION) connected at the front-end part of the sample transfer line. Mass calibration and tuning of the system in both ionisation modes are performed once per week or more often if required. The stability of the whole system is monitored daily by introducing gaseous standards (eg, acetone) at known concentrations using a gas standard generation system that additionally allows quantification calculations of target metabolites.

##### Identification of metabolites

A strategic feature of the Orbitrap is the ability to obtain tandem mass spectra (MS2) for selected ions through collision-induced dissociation. This feature significantly enhances the instrument’s chemical specificity, enabling the distinction between isomers, which is crucial for molecular identification and quantification. EBC, also collected during online breath sampling for each participant, will be analysed using standardised liquid chromatography-mass spectrometry (LC-MS) methods (reversed-phase and HILIC) and GC-MS for comprehensive metabolite coverage and chemical identification using spectral libraries.

##### Quantification of breath metabolites

Quantification of selected exhaled breath metabolites of interest is of major importance. Currently, SESI-MS is semi-quantitative and quantification is only possible for the metabolites for which the calibration curves can be obtained. Thus, to quantify detected exhaled breath metabolites, we will use a range of built-in-house or commercial systems for the controllable generation of gaseous standards in a chemical environment simulating human exhaled breath. By generating calibration curves of detected metabolites of interest using reference material, we can provide quantitative information for these specific metabolites. We can already quantify SCFAs in the gas phase (in the range of low ppt to high ppm), which are key bioactive components in the gut produced by dietary components, and the metabolism of lactose by the colon in lactose malabsorbers by colonic fermentation.^86^ This will complement the mechanistic analysis of the H_2_ breath test.

### EBC sample analysis

EBC samples will be analysed via dynamic headspace vacuum transfer in-trap extraction gas chromatography-mass spectrometry and LC-MS. GC-MS will use an Agilent 7890B GC coupled to a 5977A mass detector with an Optima 5 MS Accent column (25 m×250 µm×0.25 µm) and helium carrier gas. Column temperature starts at 35°C for 3 min, rising to 300°C at 5 °C/min over 60 min, at 0.8 mL/min flow. Electron impact spectra are collected from m/z 30–350 at 230°C. LC-MS analysis employs an Acquity UPLC system coupled with a Q-Exactive mass spectrometer. Reverse-phase separation uses BEH C18 columns (2.1 mm×150 mm, 1.7 µm) at 30°C, while hydrophilic separation uses BEH Amide columns (2.1 mm×150 mm, 1.7 µm) at 45°C, both with respective pre-columns.

### Hydrogen breath test

Exhaled breath H_2_ will be measured using a non-invasive hydrogen gas microanalyser (QuinTron BreathTracker Digital Microlyzer, model SC; QuinTron Instrument Company, Milwaukee, Wisconsin, USA). The BreathTracker has a resolution of 1 ppm H_2_ and a linear detection range between 2 and 150 ppm H_2_ with an accuracy of ±2%. It also features a two-step calibration and a purging mode that eliminates cross-interference (inter-subject contamination) and provides a stable internal baseline. The gas analyser will also be calibrated externally using a known-concentration H_2_ compressed gas standard. Breath samples (30 mL each) will be insufflated in the Breath Tracker and will be collected as CO_2_-corrected H_2_, expressed in parts per million (ppm). The maximum increase in breath H_2_ will be calculated by subtracting baseline H_2_ values from the highest H_2_ value post-lactose ingestion based on the BreathTracker readings. An increase of at least 20 ppm hydrogen between any two time points after 30 min in the study will indicate LM.^80 87^

### Gases measured by the ingestible sensor

Gas concentrations in the gut are higher than those detected in exhaled breath. To track specific gases/biomarkers produced by endogenous chemical conversions, enzymatic perturbations and the metabolic activity of the intestinal microbiota on their interaction with unabsorbed nutrients, the participants will swallow an ingestible gas-sensing capsule—Atmo Gas Capsule (Atmo Biosciences, Australia)—within 5 min following the intake of the nutritional challenges (lactose and glucose solutions).^88^,^90^ The gas-sensing capsule is a 2 cm long capsule containing miniaturised gas sensors that operate in aerobic and anaerobic conditions, a temperature sensor, a microcontroller, a radio-frequency transmitter and button-sized silver-oxide batteries. The capsule uses a membrane that allows gases to pass through and simultaneously keeps out gastric acid. The gas-sensing capsule will directly sense target gas production within the gut and evaluate the constituents (%) of H_2_, O_2_ and CO_2_, present as the capsule travels through the gastrointestinal tract (GIT). It will use O_2_ concentration to track the capsule’s location and measure gastric, small intestinal and colonic transit time. A temperature sensor will inform participants when the capsule is excreted. Data are collected every 5 min for 72 hours and are transferred from the capsule to an external portable receiver that connects and transmits the data via Bluetooth to a phone application and is transferred to the researchers via an online portal.

#### Bowel sounds measured by wearable sensors

The DigeHealth AG devices (Zurich, Switzerland) are equipped with several features designed to capture comprehensive bowel activity data: a bowel monitoring microphone, an environmental noise microphone, a photoplethysmogram sensor, gyroscopes and accelerometers. Short periods of device removal are allowed for essential hygiene (eg, showering). Participants should refrain from non-essential activities that require the device removal (eg, swimming) during the study monitoring period.

### Metabolites in urine, including lactose-derived metabolites

Lactose and its metabolites, galactose, galactitol and galactonate, will be measured in urine by targeted GC-qTOF, as this method previously demonstrated sensitivity in capturing lactose-derived metabolites,^91^ crucial for the planned mechanistic investigation. Following the published protocol,^91^ samples will be prepared to equal specific gravity to normalise concentration differences. The preparation order and GC-qTOF analysis of samples within and between volunteers will be randomised. Quality control (QC) samples and blanks will aid in data filtering and quality control. Derivatisation will involve mixing 100 µL of each urine sample with 50 µL of an internal standard solution containing isotopically labelled D-fructose and D-xylose. The two-stage derivatisation and subsequent steps will follow the HUSERMET protocol. Urine samples will be analysed on a GC 8890/qTOF 7250, and acquired GC-qTOF chromatograms will undergo untargeted metabolomics analysis for complete molecular characterisation and associations with the breath metabolome.

### Genetic analysis

We will investigate five genetic variants in the minichromosome maintenance complex component 6 (MCM6) upstream of the LCT gene associated with LP. These single-nucleotide polymorphisms (SNPs) are: −13910C>T (rs4988235), −13915T>G (rs41380347), −14010G>C (rs145946881), −22018G>A (rs182549) and −13907C>G (rs41525747). Genomic DNA will be isolated from samples collected from participants using painless buccal swabs for 15–30 s. After collection, test kits will be shipped to the central testing facility for analysis. Samples will be analysed using PCR and allele-specific primer extension to identify the polymorphisms in the MCM6 gene.

#### Metagenomic analysis

Faecal microbiota composition will be evaluated in samples collected by participants using shallow shotgun sequencing. This method, at a resolution as low as 0.5 million sequences per sample, recovers more accurate species-level taxonomic and functional profiles of the human microbiome than the widely applied 16S rRNA gene amplicon (16S) sequencing, which is only accurate to genus level, and is only moderately accurate in predicting functional profiles. The technique thus offers a pragmatic solution to accessing some of the strengths of deep whole-metagenome shotgun sequencing, which provides high taxonomic and functional resolution but is prohibitively expensive for studies with high numbers of samples. Sequencing will be carried out using state-of-the-art Illumina technology and pre-defined data processing pipelines. Collective and specific microbiota composition, alpha and beta diversity and functional differences will be investigated between the three study groups and in relation to baseline diet.

### Dietary analyses

Dietary intake will be assessed by online 24-hour recalls, supported by photo documentation, on four consecutive days (including at least 1 weekend day). Dietary recalls will be conducted using the validated myfood24 platform,^92 93^ adapted for Switzerland by integrating the Swiss Food Composition Database.^94^ All records will be verified and cross-checked by a dietitian. Foods will be grouped according to the Swiss Food Pyramid, with subgroups described by Chatelan *et al*.^95^ Daily intakes for food groups and all macronutrients and micronutrients evaluated will be extracted for further analysis. The dietary intake will be linked with lactose tolerance genotype and phenotypes to provide insights into how different relevant diet features, such as other FODMAPs and dietary fibre, influence lactose metabolism. Further, the analyses of the faecal microbiome will allow dietary data to be integrated with features of the gut microbiome, and both will be associated with the breath metabolome.

### Outcomes

#### Primary endpoints

The primary endpoint is the diagnostic performance of the Lactobreath profiles (the molecular breath profiles) for discriminating different clinical traits associated with LM after lactose intake. The choice of this endpoint allows an objective comparison with the current diagnostic measures used for LI diagnosis. It thus allows the novel test to be benchmarked against the current practice.

#### Secondary endpoints

The secondary endpoints are the clinical and metabolic traits that are associated with LM. These endpoints have been selected to allow a mechanistic understanding of the signals obtained in/as part of the Lactobreath profiles. The secondary endpoints are also important for making the link with existing diagnostic methods used in the assessment of food intolerances.

### Statistical power calculation

Power analyses for human intervention studies conducting metabolomics analyses have been published,^81^ but the applicability of this method still needs to be improved. The Lactobreath project consequently bases the targeted number of participants for each group on previously published evidence by research groups in nutritional metabolomics, including Agroscope.^96^,^98^ Among others, these studies have demonstrated that 12–15 subjects per intervention group are already sufficient to discover, among the thousands of metabolites measured, dozens if not hundreds of postprandial metabolites responding to the intervention differentially with statistical significance after correction for multiple testing. In addition, our recent pilot study on 11 participants for the characterisation of the human postprandial breath metabolome following a nutritional challenge with a standardised meal revealed thousands of features detected using our SESI-MS, including 265 structural candidates for the intervention-related metabolites.^78^ Postprandial metabolomics analysis has also shown its potential to identify distinct human metabotypes, monitor time-resolved metabolic changes underlying a disturbance (eg, stress, starvation, food intake) and regain homeostasis in a group of 70 participants.^99^

### Statistical analysis

#### Primary endpoint

Supervised machine learning algorithms (such as random forest and partial least squares discriminant analysis) will be applied based on Zheng *et al*^100^ and will identify molecular breath profiles that discriminate different clinical traits associated with LM based on the levels of metabolites in the exhaled breath after the intake of lactose. Models will be created to explore separately the breath profiles that are discriminated for each trait of LM, including the response to the LHBT, polymorphisms for LP, lactose-derived metabolites in urine and the GI symptoms of LI taken individually and collectively. To reduce overfitting and deliver reliable estimates of the models, we will apply machine learning with voting, a strategy for classification based on selecting the output with the most classes. Clinical traits will be modelled as either categorical variables, continuous variables or both, where appropriate. This analysis will be conducted first on a subset of 50% of the recruited participants to allow the most predictive metabolites to be identified within the timeframe of the study. The subset will be balanced to represent the three test groups. The same analysis workflow will later be applied to the whole cohort, with 20 participants (stratified for group) removed from all modelling to allow for internal validation of the models. The best profiles (Lactobreath profiles) that associate the breath exhalome with each of the clinical traits of LM will be tested in the 20 remaining participants to assess their validity. Further, to consider the specificity of the Lactobreath profiles for lactose metabolism, the profiles will be evaluated in the participants assigned to the glucose test with additional comparisons between intervention types (glucose vs lactose) across all three participant groups. The molecular profiles with the best performance for predicting LM clinical traits will be retained to determine the identity and quantification of the molecules composing the selected profiles.

The diagnostic performance of the lactose-induced breath profiles identified for the clinical traits will be assessed using the following parameters: sensitivity, specificity, positive predictive value, negative predictive value, the likelihood ratio of a positive test, likelihood ratio of a negative test as described by Beyerlein *et al*^17^ and Jellema *et al*.^81^ For each parameter, the novel breath profiles will be compared with the reference diagnostic tool. The participants are grouped by LHBT and genetic test results as these are commonly used in clinical practice for LI diagnosis and are, therefore, suitable for evaluating the performance of the breath biomarkers. The level of significance will be two-sided (α=0.05). Corrections for multiple testing will be made using the false discovery rate.^101^

Analysis populations: the analysis populations are defined by the criteria used to select the three study groups and the two assigned carbohydrate interventions, resulting in six groups for analysis.

#### Secondary endpoints

To explore the mechanisms underlying the Lactobreath profiles, first, unsupervised, integrative analysis (such as correlation-based tools like regularised canonical correlation analysis) will be used to link the metabolic traits (ie, urinary metabolome, lactose-derived colonic gases) and clinical traits with specific features of the Lactobreath profiles. In the second step, integrative data analysis tools, such as similarity network fusion tool, and Data Integration Analysis for Biomarker discovery using Latent variable approaches for ‘Omics’ (DIABLO; MixOmics)^102 103^ will be used to combine breath markers and pairs of metabolic/continuous clinical traits with respect to categorical clinical traits to explore the potential of these traits for improving the predictivity of the Lactobreath markers.

Statistical analyses will be conducted using R and Python programming environments. The final trial report will describe and justify any deviation from the original statistical plan. The stopping rules for the study are a single serious adverse event.

### Missing data and drop-outs

Missing data will be managed by multiple imputations using the moving average method for the intervention postprandial measurements. Missing data for the baseline measures will be handled by imputing the mean of all baseline measurements available for the participant. Additional subjects will be recruited from the group of participants who complete the home survey, should the number of participants completing the lactose and glucose tests fall below the targeted numbers due to drop-outs.

### Patient and public involvement

Patients or the public were not involved in the design, or conduct, or reporting, or dissemination plans of our research. All results from the study will be published publicly and accessible without restrictions, in accordance with the Swiss National Science Foundation open access policy. The accepted version will be deposited in the ETH Research Collection (www.research-collection.ethz.ch), the so-called ‘green way’ for open access. The microbiota data will be published at the European Nucleotide Archive (https://www.ebi.ac.uk/ena/browser/home) after all relevant data have been published. Details of the study design will be available through the phenotype database. All other data will be accessible from within our organisation, ETH Zurich. If an external scientist wishes to access the data, there are straightforward options to do so (eg, setting up a guest account at ETH that allows access via a virtual private network (VPN) account). Such access will be granted on reasonable request.

## Ethics and dissemination

### Ethics approval

The Lactobreath study has been approved by the Ethics Committee of the Canton of Zurich, Switzerland (#2023-01639).

### Data statement section

All data will be coded with an internal participant ID code initiated at enrolment. The participant identification list will be stored securely and separately from all biological material and study data. This list will be destroyed after the legally prescribed retention period (10 years). Access to all computers storing study data is protected by a password with additional internal restrictions limiting access to project files to personnel requiring access to the project data. Data generated on online platforms (eg, 24-hour recalls) will be entered using participant codes and securely stored prior to transfer to local servers.

Coded genetic data will be used to assess selected penetrant SNPs for lactase persistence. This data will be collected and handled according to state-of-the-art genetic testing to ensure confidentiality, in line with ‘Verordnung über genetische Untersuchungen beim Menschen’.

Biological material will be identified by the unique participant code to ensure traceability. Biological material is appropriately stored in a restricted area only accessible to authorised personnel to prevent unauthorised disclosure, alteration, damage or loss of biological material. Microbiota data pre-processing will apply a standard pipeline to remove any contaminating human reads before sharing data files.

### Data availability

The original data generated by the study will be accessible in a curated data archive at ETH Zurich (https://www.research-collection.ethz.ch).

## Discussion

The Lactobreath study uses state-of-the-art real-time breath metabolomics, suited for sensitive, non-invasive measurement of breath biomarkers in a controlled nutritional intervention that offers extensive and comprehensive participant characterisation for clinical and metabolic features relevant to lactose metabolism. The holistic approach defined in the Lactobreath study aims to address and explore some of the challenges in the diagnosis of food intolerance by capturing metabolic processes and physiological variables that influence clinical phenotypes. Indeed, the Lactobreath breath profiles focus on assessing clinical symptoms in the context of multiple existing diagnostic tools as well as physiological factors (eg, gut microbiota, GI transit) that are known to influence food intolerance symptoms.

In the clinical field, there is currently a gap in the accurate detection of exhaled breath biomarkers for medical diagnostic tests. The Lactobreath study, with the extensive clinical characterisation of the subjects, aims to support the development of a diagnostic test for LM and LI based on exhaled breath metabolomics, providing a proxy for the subjective GI symptoms experienced by humans. The Lactobreath study will serve as proof of concept to extend the use of breath metabolite profiles to the more holistic topic of the diagnosis of food intolerance and its dietary management with low FODMAP consumption.

Notably, the reproducibility of the findings in different populations will need to be confirmed in a second study. The priority in this study is to confirm the proof of principle that dietary response can be distinguished via breath biomarkers. In this context, selecting participants from a limited population sample is important to control other factors that could confound the findings (eg, ethnic group). Moreover, the specificity of discriminating biomarkers to LM will need to be confirmed in a patient setting with other GI disorders.

## Supplementary material

10.1136/bmjopen-2025-107256 online supplemental file 1
