# Supplementary material for: Exploring exhaled breath biomarkers for lactose intolerance diagnosis: the Lactobreath pilot study protocol
Source: BMJ Open. 2025 Aug 3;15(8):e107256. doi: 10.1136/bmjopen-2025-107256 (PMC12320064; doi:10.1136/bmjopen-2025-107256)
Supplement: online supplemental file 1 [file bmjopen-15-8-s001.docx]

Application to take part in medical research

Study name : Lactobreath: A pilot study to diagnose lactose intolerance based on the exhaled breath metabolome

Study short name : Lactobreath

Dear Sir or Madam,

We would like to introduce you to the Lactobreath Study and invite you to take part. Before a new diagnostic test can be used, research is needed to ensure the test works well.

In medicine, such research is called a **clinical study**. In this study, we want to find out whether we can diagnose food intolerances, which you may have experienced, by using biomarkers we can detect in breath. This is why we are asking you if you would like to take part in this study.

Your participation is voluntary. This **information document** is intended to help you make your decision. You can ask any questions you may have during a **meeting with the study researcher**. This person is responsible for monitoring the participants in the study. If you agree to take part in the study, please sign the **declaration of consent** at the end of the document. By signing, you confirm that you have read and understood the information provided. If there is anything you do not understand, please do not hesitate to ask the study researcher for clarification.

The information and consent form consists of four parts:

**Part 1 The essentials- a summary**

**Part 2 Detailed information about the study**

**Part 3 Data protection and insurance cover**

**Part 4 Declaration of consent**

**Part 1** gives you a general overview of the study. In **part 2**, we explain in detail how the study will be conducted and the context in which it will take place. **Part 3** contains information about data protection and insurance cover. By signing the consent form at the end of the document, **part 4**, you certify that you have understood all the information and agree to take part.

This study is initiated by ETH Zurich. This institution is called the sponsor. The sponsor assumes responsibility for the management and financing of a study.

The contact person for this study is :

Name Prof. Dr. Daniel Pohl
Address University Hospital Zurich, Raemistrasse 100, 8091 Zürich
Telephone number +41 44 255 85 48

Email [Daniel.Pohl@usz.ch](mailto:Daniel.Pohl@usz.ch)

Part 1 :
The essentials- a summary

Vor allem ab Phase-3-Studien (IMP) oder konfirmatorischen MD-Studien.

# Why are we conducting this study ?

There are currently different methods that can be used for diagnosing food intolerances like lactose intolerance. Some methods are very invasive while other methods give results that do not inform the physician about important aspects of lactose metabolism.

In this study, we examine how markers in the breath can be used to understand lactose digestion, metabolism and intolerance, and to assess the performance of such markers for diagnosing lactose intolerance. You can find out more about the scientific background to the study in chapter 4.

# What do you have to do if you participate?

Your participation will last approximately 4 weeks. We will invite you to 1 intervention visit and 1 close-out visit as part of the study. The intervention visit lasts approximately 8 hours. The close-out visit lasts approximately 1 hour.

If you decide to take part, you will be part of either an intervention group or a control group. You will be randomly assigned to one of the two groups. You will not know which group you belong to. In the intervention group, you will receive the lactose solution. In the control group, you will receive a glucose solution. Both groups will have to adhere to strict dietary restrictions for three days before the intervention day.

You can find out more about the study process and procedures in **chapter 5**.

# What are the benefits and risks linked to participation?

## Benefits

There is no direct benefit from taking part in this study. However, your participation may help others who suffer from food intolerances. The possible benefits concern learning more about food intolerances and receiving information about how your body responds to lactose consumption.

## Risks

The Atmo Gas Capsule that will be used to monitor gases in the gut is not yet certified in Switzerland.

We may not yet know all the risks and side effects of the Atmo Gas Capsule. So far, there are no known side effects and the following risks are known:

- Capsule retention: this means that the gas-sensing capsule is not eliminated from the body within 14 days of ingestion

The risks of using the gas-sensing capsule are reduced by using the capsule only under the conditions recommended by the manufacturer:

- The gas-sensing capsule should not be used for people with certain diseases and conditions. These diseases and conditions are all listed in the study exclusion criteria and are assessed during the inclusion visit.
- The gas sensing capsule should not be used with certain medications. Volunteers using these medications cannot participate in the study.
- Dietary restrictions apply before and after the ingestion of the capsule.
- Participants should not undergo an MRI examination while the study is in progress and the capsule is not excreted. Participants should not undergo an MRI within 3 months of ingesting the gas-sensing capsule if the excretion of the capsule is not confirmed. In this case an x-ray would be required to confirm the elimination of the capsule from the body.

More information on risks and constraints can be found in **chapter 6**.

Part 2 :
Detailed information about the study

# Scientific background

## Context: why are we conducting this study?

Food intolerances are a frequent condition in adults affecting 15-20% of the population.

When you have lactose intolerance you typically suffer from bloating, abdominal discomfort, flatulence and diarrhoea when you consume lactose in the diet. The condition lactose intolerance is generally treated with dietary therapy following diagnosis by restricting consumption of lactose and foods containing lactose, or using synthetic lactase formulations when consuming lactose-containing foods.Research has already been carried out in humans on lactose intolerance. Studies conducted to date have shown that symptoms of lactose intolerance are most commonly caused by the absence or low levels of an enzyme called lactase that is necessary to digest lactose. The production of this enzyme in adults depends how certain information present in our genes.

We also know that lactose intolerance can be diagnosed by measuring specific molecules in blood, urine or breath after a solution of lactose is consumed. Lactose intolerance can also be diagnosed by assessing the genes involved in producing lactase or by directly measuring lactase in the gut.

We don't know why some tests for lactose intolerance do not match the symptoms of the patient. In particular, we lack data on why some people with lactose intolerance are very sensitive to lactose while others can consume lactose with few symptoms.

In this study, we are therefore examining whether multiple breath biomarkers can be used for the diagnosis and understanding of lactose metabolism in the case of lactose intolerance. We will compare the breath biomarkers to other diagnostic tests and assessments to diagnose lactose intolerance.

The Atmo Gas Sensor used to track gut gases during the lactose test is not yet authorised in Switzerland but it has undergone safety testing in human studies. Over 560 patients have used Atmo Gas Sensor in clinical trials with no serious adverse events. Only when the performance of the medical device has been scientifically studied can it be authorised in Switzerland.

In addition, we are studying how the gut microbiota (defined as the collection of microorganisms that colonise the gastrointestinal tract) and diet can affect lactose metabolism. The results of this study should show why people experience different symptoms with lactose intolerance.

## Structure of the study : how do we do it ?

In our study, we use two screening tests that eligible participants must complete at home. These tests allow us to group participants by how they metabolize lactose.

- A lactose tolerance test: a solution of lactose will be consumed and a questionnaire on lactose intolerance symptoms will be completed.
- A genetic test for lactase persistence (the ability to digest lactose as an adult): a sample of saliva will be collected by the participant and sent by post to the research centre for the measurement of genes that are necessary for lactose digestion.

Depending on the results of the two tests combined, participants will be included or excluded from the study.

Selected participants are randomly assigned to groups, a process known as randomisation. This method is important for obtaining reliable results. Each group receives a different treatment. In our study, there are two groups:

- The **lactose group** (intervention group) receives a lactose solution (25 g of lactose dissolved in 1.5 L water)

- The **glucose group** (control group) receives a placebo, i.e. a glucose solution that should not provoke symptoms of lactose intolerance (13 g of glucose dissolved in 1.5 L water)

This is a so-called "double-blind" study, which means that none of the people involved in carrying it out know who is in which group: neither the participants nor the researchers know the allocation between the groups. This is why it is called "double blind". This method is chosen so that participants have as little influence as possible on the results of the study. Randomisation and double-blinding allow us to objectively assess the response to the dietary substance being tested.

## Regulation of scientific research involving humans

We carry out this study in accordance with the laws in force in Switzerland (law relating to research involving humans, data protection laws). In addition, we comply with all internationally recognised guidelines. The study has been reviewed and authorised by the relevant ethics committee.

Our study is a national study. There are 120 participants in Switzerland.

You can also find a description of this study on the website of the Federal Office of Public Health, at www.kofam.ch, under the registration number SNCTP 000005682 or the BASEC number BASEC 2023-01639

# Study procedures

## What must you do if you participate in the study?

Participation in the study is voluntary and lasts for approximately 4 weeks. You must keep to the schedule of visits (🡪 chapter 5.2) and follow all the instructions given by the research team.

You must inform the research team

- if your health changes, for example if you feel less well or if you develop new health problems; you must continue to inform them if you withdraw from the study (🡪 chapters 5.3 and 5.4);
- if your lifestyle changes, for example you begin smoking

You should also consider the following points:

- You must not become pregnant during your participation (🡪 chapter 5.5).
- You must follow the study dietary restrictions for three days before the study visit and intervention day
- You must not eat or drink anything except the sugar solution (either lactose or glucose solution) and water given to you by the study researchers on the morning of the study intervention and during the intervention.
- You must not have an MRI examination until we confirm that the gas-sensing capsule has been recovered.

## What happens during study visits ?

You will come to the study centre twice. The intervention visit lasts approximately 8 hours. The close-out visit lasts approximately 1 hour.

Here’s what we do to prepare for the intervention visit:

- Two to three weeks before the study visit day, we will assess your microbiota and diet. To do this at home, we will provide you with the necessary materials today:
  - You will complete four dietary assessments to record all food and drink consumed on three sequential days
  - You will collect three stool samples, ideally on three sequential days, starting from the day after the first dietary assessment
- You will follow a strict diet for three days before the study visit: this means you will follow the diet defined by the study researchers which will avoid all foods that can cause symptoms of food intolerance. All food will be provided to you for this period. No alcohol is permitted during these three days before the study visit.
- You will be fasted on the morning of the study visit except for 300 mL of water.

Here's what we do during the intervention visit:

- We answer your questions.
- We ask you questions about your health, any gastrointestinal symptoms (by questionnaire) and confirm your vital signs before starting the test
- We ask questions about your diet and food records.
- We will make some baseline breath and urine measurements before starting the test.
- We confirm your height and body weight measurements.
- We will start the test by giving you a sugar solution (either lactose or glucose in 150 mL water) to consume.
- We will give you a gas-sensing capsule to swallow immediately after the test starts to monitor gases in the gut during the test. Information from the capsule is collected by a capsule receiver that is worn on a belt. You must keep the receiver within a 2- meter distance from your body during the test and until the capsule has been excreted.
- Thirty minutes before the start of the test, you will be instructed to attach two wearable devices around your abdomen to record digestion sounds over the test period and until 9 h after the beginning of the test.
- We measure the volatile organic compounds (VOCs) and hydrogen found in the breath you exhale at multiple time points for 6 h after the sugar solution is consumed.
- We measure metabolic degradation products that are present in your urine at multiple time points for 6 h after the sugar solution is consumed.
- We assess your tolerance of the sugar solution by a questionnaire that you will complete at multiple time points for 9 h after the sugar solution is consumed.
- At the end of the 6 h test, you will receive a standardized meal.

These examinations enable us to assess how your body responds to consuming lactose or glucose. They will tell us about the chemical composition of your breath and urine after consuming lactose, and help us identify specific metabolic profiles associated with gastrointestinal symptoms related to lactose intolerance. The gas-sensing capsule will measure intestinal gases as it moves through the GI tract which will provide us information about lactose metabolism.

For those receiving the lactose solution, these measurements could inform us about whether you have signs of lactose intolerance or lactose malabsorption.

We arrange the visits together. Should you have to reschedule a visit for important reasons, please inform us as soon as possible.

## When does participation in the study end ?

Your participation lasts for up to 4 weeks and ends after the confirmation of the elimination of the gas-sensing capsule. You can stop taking part at any time before this date (🡪 chapter 5.4). You do not need to justify your decision. If you wish to end your participation, please inform the study researcher.

In this case, we will carry out a final examination as part of the study, for your own safety. We will ask you to return the capsule receiver and belt, as well as the two wearable devices and their belts.

If you stop the study before the planned date, please continue to inform the investigating doctor if your health changes, for example if you feel less well or if any new problems appear. If you withdraw from the study, we will still be able to analyse the data and samples collected up to that point (e.g. breath samples).

We may also have to exclude you from the study earlier. This may be the case, for example if you are unwell during the study period.

## What happens if you do not want to participate ?

If you do not take part in this study, there is no requirement to justify your decision and you are free to withdraw your interest. If consent is withdrawn, all data already collected and samples taken will still be evaluated as part of the study. The data will remain encrypted in the study documents.

## Pregnancy

The gas-sensing capsule used in this study is not yet certified in Switzerland. It could require investigations that could be dangerous and harmful to an unborn child. Pregnancy and the associated physical changes could falsify the study data.

You may therefore not have children while you are taking part in the study. You should discuss these questions with the study researcher.

### For women who are considering a pregnancy

You must avoid becoming pregnant while taking part in the study. You must inform your partner(s) that you are taking part in this study. Before ingesting the gas-sensing capsule, you will take a urine pregnancy test.

During your participation in the study, you must use a very effective method of contraception:

1. a method that suppresses ovulation, either in the form of a tablet ("pill" / "mini-pill"), injection, implant (under the skin), patch or vaginal ring or,

2. a hormonal or copper coil.

You must continue to use these methods of contraception for until the gas-sensing capsule has been recovered or confirmed as excreted. If you nevertheless become pregnant during the study, you must inform the investigator immediately. He/she will discuss the next steps with you and your partner.

# Risks, restrictions and side effects

## What are the risks and restrictions associated with the study ?

Taking part in this study involves risks and restrictions. We already know about some of the risks, while others are still unknown. This uncertainty is not unusual in a study context. You will find a list of the most frequent and serious risks in **chapter 6.2**. Many side effects can be treated medically. We will keep you informed during the study of any new knowledge about risks and side-effects.

A new medical device may entail risks we do not yet know about.

The medical device has been used by few people.

The medical examinations we carry out as part of the study also involve risks. Some examinations are linked to the standard protocol for diagnosis of lactose intolerance. You will find a list of the risks associated with these examinations in **section 6.3.**

## Most common and frequent risks associated with the Atmo Gas Sensor

Here you will find information about the most common and most serious side effects that we already know about.

We use the following categories to describe them:

| Very common | The side effect occurs in more than 10 in 100 people (more than 10%). |
| --- | --- |
| Common | The side effect occurs in 1 to 10 people out of 100 (1%-10%).’ |
| Occasional | The side effect occurs in 1 to 10 people out of 1000 (0.1%-1%). |
| Rare | The side effect occurs in 1 to 10 people in 10,000 (0.01%-0.1%). |
| Very rare | The side effect occurs in less than one person in 10,000 (less than 0.01%). |

Common side effects include:

- Bloating
- Abdominal pain

## Risks and restrictions associated with the examinations conducted during the study

We carry out various medical examinations for this study ((🡪 chapter 5.2). These examinations are established procedures. Nevertheless, they may involve risks and restrictions, i.e. they may be unpleasant or have undesirable side effects. In this study, the risks and restrictions are as follows:

- Lactose test : the lactose test requires the ingestion of a single lactose solution. In the case of lactose intolerance, symptoms of lactose malabsorption may arise including: bloating, abdominal discomfort or pain, flatulence, and diarrhoea.
- Dietary restrictions: before the day of the study intervention, you will be asked to follow a specific diet for three days. During this time you will only be allowed to consume foods defined by the study researchers and no alcohol. On the day of the test you must come to the test centre in a fasted state (i.e. consuming no food or drink except for water in the 12 hours preceeding the test). One 300 mL glass of water should be consumed in the morning of the test. During the measurement period you will only be allowed to consume the test solution and water. It is important to following the dietary restrictions to ensure that the test results are not affected by other foods in your diet but it may be constraining for you.
- **Pre-test hygiene restrictions: on the day of the study intervention you may brush your teeth but using only water and no toothpaste.**
- Atmo Gas Sensor restrictions: Before the confirmed excretion of the gas-sensing capsule, **participants must not undergo an MRI examination unless an x-ray is performed to confirm that the capsule has left the body.** Participants should also avoid engaging in strenuous exercise while the capsule is being used and until it is confirmed as excreted.
- DigeHealth wearable devices: The main known risk is minor skin irritation due to prolonged contact between the skin and the device. Only a small percentage of participants may experience such discomfort, and they are free to remove the device should this occur.

# Funding and compensation

This study is entirely funded by the Swiss National Science Foundation (SNSF).

The researchers taking part in the study are not receiving any direct financial benefit.

You will receive the following compensation if you take part in this study:

- 300 CHF upon the completion of the full study protocol.

If the study intervention is started but stopped for any reason, you will be compensated *pro rata* for the time spent in the laboratory and for travel costs.

If you are excluded after the screening test, only travel costs for attending the laboratory will be covered.

There are no additional costs to you or your health insurance for taking part in the study. We will reimburse you for travel expenses incurred as a result of your participation (including public transport second class tickets).

The results of this study may contribute to the marketing of a medical device. Your participation does not give you any rights regarding its commercial exploitation.

# Results of the study

The results that concern you personally are communicated to you by the investigator. Sometimes results are discovered by chance. For example, there may be results about your metabolic status. We inform you if these discoveries are important for your health.

If you do not wish to receive this information, please speak to the study researcher.

In addition to results for the individual, the study will produce collective results based on data from all the people taking part. These could include new knowledge about food intolerances (🡪 chapter 4.1). These results do not concern you or your health directly. If you wish, the investigator will provide you with a summary of the overall results at the end of the study.

Part 3 :
Data protection and insurance coverage

# Protection of data and samples

We will protect your data (e.g. data from the study assessments like body weight and height) and your samples (e.g. your urine samples). Swiss law lays down strict rules on data and sample protection.

## Coding of data and samples

Every study generates data from examinations and assessments (e.g. body composition, urine composition values, bowel sounds). This data is recorded in coded form, usually electronically. Coding means that personal information is stored *separately* from other data, in the form of a list that identifies each person with a unique code. This means that your name, date of birth or address do not appear directly with the other data collected. This list remains for 10 years at the ETH Zurich institution. Nobody else receives it.

Environmental sound data collected by the wearable bowel sounds devices will be encrypted at the point of data acquisition and will only be exploited to support the assessment of bowel sounds. As soon as the data is processed the environmental sound data will be destroyed. If the participant does not wish the devices to record the environmental sounds during the 9h of monitoring they may remove the devices.

At the end of the legally prescribed retention period, your data will be irreversibly anonymized. This means that it will no longer be possible to re-identify you without disproportionate effort. This involves several de-identification measures, including the destruction of the code and the list.

When we transfer data to specialists who carry out other analyses, it is always coded and your personal data is protected. The same applies when data is transferred abroad.

All biological samples (e.g. urine samples) are also coded in this way. Your personal data is therefore protected when we send samples for laboratory analysis. Data and samples are always coded at the laboratory.

## Security of data and samples during the study

The sponsor ETH Zurich is responsible for the security of your data and samples in this study. The sponsor will ensure compliance with applicable laws, such as data protection laws. This also applies when (coded) data or samples are sent for analysis to countries with less stringent data protection laws. This is how the study sponsor protects your data:

In this study, your data is captured and transferred electronically. The data is primarily stored on a server in Switzerland. Data on dietary intake is stored during the study in encrypted form on a server in the UK. At the end of the study all data will be transferred to a server in Switzerland. Nevertheless, the risk of unauthorised persons gaining access to your personal data cannot be entirely ruled out (e.g. risk of "hacking").

Your samples are also used in this study for genetic analysis of your hereditary information (in what is known as deoxyribonucleic acid = DNA). The results obtained are "genetic data" that are stored on a server in Canada before transfer to a server in Switzerland. This data is also protected. However, the risk of your genetic data being used to identify you cannot be entirely ruled out.

## Security of data and samples at the end of the study

When the study is over, the sponsor continues to ensure the security of your data and samples. By law, all study documents, such as data collection forms, must be kept for at least 10 years. If sample residues remain at the end of the study, we will destroy them.

Once the study has been completed, the results are generally published in scientific journals. To do this, the data is sent in coded form to other specialists so that they can review the publication. These data cannot be reused for research purposes.

## Rights of consultation during inspections

The implementation of this study may be subject to checks and inspections. These inspections are carried out by authorities such as the competent ethics committee or the Swissmedic authorisation authority, or by foreign authorisation authorities. The sponsor must also carry out checks to guarantee the quality of the study and its results.

For these checks and inspections, a small number of specially trained people have access to your personal data and medical records. The data is therefore not coded. The people who consult your uncoded data are bound by professional confidentiality.

# Insurance cover

You are covered by insurance if you suffer damage as a result of the study - i.e. as a result of the medical device or the intervention protocol. The procedure is governed by law. The sponsor has taken out insurance with Baloise Versicherung AG (Hauptsitz, Aeschengraben 21, Postfach, 4002 Basel). If you believe that you have suffered damage as a result of the study, please contact the study researcher or the insurance company directly.

Part 4 :
Declaration of consent

Please read this form carefully. Do not hesitate to ask us questions if you do not understand something or if you need clarification. Your written consent is required to participate.

### Declaration of consent for the participation in the Lactobreath study

| **BASEC number** |  |
| --- | --- |
| **Study name** | Lactobreath: A pilot study to diagnose lactose intolerance based on the exhaled breath metabolome |
| **Study short name** | Lactobreath |
| **Responsible institution** (sponsor and address) | ETH Zürich, Vladimir-Prelog-Weg 3, CH-8093 Zürich, Schweiz |
| **Location of study** | Department of Chemistry and Applied Biosciences at ETHZ |
| **Responsible study researcher on-site** | Dr Stamatios Giannoukos |
| **Participant** Full name in block capitals : Date of birth : |  |

- I have received oral and written information about the study from the undersigned study researcher.
- The study researcher has explained to me the purpose, procedures and risks of the study.
- I am voluntarily taking part in the study.
- I have had sufficient time to make my decision. I will keep the written information and receive a copy of my written declaration of consent.
- I can end my participation at any time, I don't need to justify it. The data and samples collected until this point will be stored and will be analysed as part of the study.
- The investigator may exclude me from the study at any time in the interests of my health.
- I understand that my data and samples will only be transferred (abroad) in encrypted form. The sponsor will ensure that data protection is complied with in accordance with Swiss standards.
- I will be informed of results and/or incidental findings that directly concern my health. If I do not wish this, I will inform the investigator.
- The relevant specialists from the Ethics Committee and the medicines control authority, *Swissmedic,* may consult my uncoded data for control purposes. All these people are bound by professional confidentiality.
- The institution's civil liability insurance Baloise Versicherung AG covers any damage.

| Place, date | Full name of participant in block captials  Signature of participant |
| --- | --- |

**Declaration of study researcher:** I hereby declare that I have explained the nature, importance and scope of the study to the participant. I declare that I have fulfilled all my obligations in relation to this study under Swiss law. If, during the course of the study, I become aware of any factors that might affect the participant's willingness to take part in the study, I undertake to inform him/her immediately.

| Place, date | Full name of study researcher in block capitals  Signature of study researcher |
| --- | --- |
